# The Impact of the Telephone Crisis Support Role on the Mental Health of Workers in the Psychosocial Support Line “10306”

**DOI:** 10.1192/j.eurpsy.2025.1643

**Published:** 2025-08-26

**Authors:** E. Denazi, T. Skali, M. Theodoratou, V. Sapouna

**Affiliations:** 1 National & Kapodistrian University of Athens, Department of Psychology; 2 School of Medicine, National & Kapodistrian University of Athens; 3 Project Scientist in Charge of RRF “Helpline 10306”, Athens; 4 Social Sciences, Hellenic Open University, Patras, Greece; 5 Psychology, Neapolis University Pafos, Pafos, Cyprus

## Abstract

**Introduction:**

Workers on helplines such as the “10306 Support Line” play a critical role in offering confidential, empathetic support to individuals in crisis, providing a secure environment for those grappling with anxiety, depression, or suicidal ideation. However, the high level of responsibility, extended hours, and often distressing nature of the calls can place substantial strain on the mental and physical health of these support line professionals.

**Objectives:**

The primary aim of this study is to examine the psychosocial risk factors and resulting physical and psychological impacts on employees of the “10306 Psychosocial Support Line”, with the intention of contributing to strategies for managing employee challenges, preventing stress-related symptoms, and promoting best practices for the mental and physical wellbeing of staff.

**Methods:**

This cross-sectional quantitative study will involve all health professionals working on the 10306 Support Line, using survey methods to evaluate psychosocial and physical impacts. The following validated psychometric tools were used:

- Copenhagen Burnout Inventory (CBI): measuring burnout levels and the emotional toll associated with support line work.
- Sense of Coherence Scale (SOC): assessing individuals’ capacity to manage stress and maintain well-being.

**Results:**

The study results indicate burnout levels among “10306 Support Line” workers. CBI scores reveal that 55.3% of participants experience personal burnout, 47.4% report work-related burnout, and 39.5% face burnout related to caller interactions. A strong negative correlation was found between burnout and the Sense of Coherence (SOC) scores, suggesting that a higher SOC is associated with reduced burnout. Additionally, younger participants reported higher personal and work-related burnout levels than their older counterparts, highlighting age as a potential risk factor for increased burnout symptoms among support line professionals.

**Conclusions:**

The high prevalence of burnout among 10306 Support Line employees highlights a critical need for ongoing mental health support and targeted stress management. Strengthening the Sense of Coherence (SOC) and addressing age-related risk factors may enhance resilience and well-being among support line professionals, supporting both their health and the quality of psychosocial care provided to those in need.

**Disclosure of Interest:**

None Declared

